# A scoping review and thematic analysis of policy implications of novel and emerging nicotine and tobacco products regulation

**DOI:** 10.3389/fpubh.2026.1786077

**Published:** 2026-03-13

**Authors:** Yoojin Choi, Sujin Choi

**Affiliations:** 1 College of Health Science, Korea University, Seoul, Republic of Korea; 2 Department of Nursing, School of Medicine, Soonchunhyang University, Asan, Republic of Korea

**Keywords:** compliance, nicotine products, public health, restriction, tobacco products

## Abstract

**Background:**

This scoping review aimed to examine policy implications from studies investigating the effectiveness of policies related to novel and emerging nicotine and tobacco products (NENTPs) and to identify commonalities in policy implications for NENTPs through thematic analysis.

**Methods:**

This scoping review involved a comprehensive search across the following four databases: PubMed, Embase, Web of Science, and CINAHL. The latest search was on March 15, 2025. The Preferred Reporting Items for Systematic Reviews and Meta-Analyses extension for Scoping Reviews guidelines were followed. Studies were included if they examined the effectiveness of NENTPs-related policies. After screening titles, abstracts, and full texts, 21 studies were selected and analyzed based on the framework to derive policy implications.

**Results:**

Most studies were conducted in high-income countries. Thematic analysis yielded the following two analytical themes: (1) regulatory governance and policy system architecture for NENTPs control, which included three descriptive themes—namely, regulatory spillover control in the national context, evidence-informed policy transition and regulation, government monitoring and compliance assurance; and (2) exposure reduction at the population level and societal regulation of NENTPs use, with four descriptive themes—namely, product attribute restrictions as an initiation control, regulation of access to retail stores and exposure to youth, economic restraint through tax increase, and customized communication for policy education.

**Conclusion:**

This study highlights that effective legal regulation of NENTPs requires not only regulatory governance and policy systems but also population-level regulatory mechanisms that reinforce the legal foundation. Our findings provide timely, integrated insights for policymakers by examining commonly shared perspectives among policy researchers.

## 1 Introduction

The market for novel and emerging nicotine and tobacco products (NENTPs) —defined here as non-combustible alternatives such as e-cigarettes, heated tobacco products, and nicotine pouches—, which utilize innovative technologies, is expanding to preserve long-term profits (1). Companies continue to launch nicotine and tobacco products of various types and with novel design features that are not covered by existing tobacco product regulations (2). The greater the variety of NENTPs, the more likely they are to become the subject of intense debate and regulatory complexity (3, 4). In particular, some products are marketed as alternatives to conventional tobacco, emphasizing that they are not combustible and making sometimes unproven claims of reduced harmfulness (5). However, they are not free of absolute risk (6), raising public health concerns.

A representative international tobacco control treaty is the Framework Convention on Tobacco Control (FCTC), which outlines several strategies necessary for countries worldwide to reduce tobacco consumption and smoking rates (7). While legal regulations are being established and strengthened in the FCTC’s participating countries, tobacco companies tend to pursue complementary interests in less-regulated countries by exploiting regulatory weaknesses, shifting their marketing and sales strategies from countries with strict controls to those with more lenient policies (8).

Globally, regulatory approaches to NENTPs vary due to differing public health priorities, cultural contexts, and policy environments (9, 10). According to a World Health Organization (WHO) report (11), 85% of high-income countries have implemented e-cigarette regulations or sales bans. In comparison, approximately 40% of middle-income countries and approximately 80% of low-income countries have not implemented regulatory action against e-cigarettes. Furthermore, the reduction of smoking rates in low- and middle-income countries has been inconsistent and slower than expected, with limited research to guide priority-setting (12).

In addition to regulatory differences across countries, the use of NENTPs by both youths and adults has emerged as a global public health issue (13, 14). According to the US National Youth Tobacco Survey in 2022, 9.4% (2.55 million) of middle and high school students used e-cigarettes, making them the most commonly used nicotine products (15). In response, numerous studies have assessed the effectiveness of regulatory policies (16) in preempting the market entry of NENTPs (17) and in establishing effective legal and institutional frameworks.

Governments have introduced diverse policy interventions in each country. Many of these policies are based on regulatory mechanisms and options specified in MPOWER (18), a WHO framework for tobacco control that includes product classifications, prohibitions, component bans, taxation, age restrictions, and marketing restrictions (19). Unlike the traditional cigarette market, NENTPs rapidly evolve through technological innovation and require a robust governance system for tobacco monopoly (20). Furthermore, if stringent regulations, are implemented, users are likely to migrate to new products (21, 22), necessitating an integrated restriction strategy differentiated from conventional cigarettes. Therefore, NENTPs regulations should be reassessed and supported by analyses from the integrated perspective.

However, studies on NENTPs policies have primarily evaluated the effectiveness and impacts of specific products, such as e-cigarettes (20), flavored electronic nicotine delivery systems (ENDS) (23), using systematic analyses or scoping reviews. In previous studies evaluating the effectiveness of NENTPs policies, researchers frequently offer policy suggestions, but no research has synthesized these suggestions into integrated policy implications. Therefore, this study aims to derive common policy implications from previous research on NENTPs through a scoping review and thematic analysis. This study findings are expected to provide integrated policy recommendations for policymakers and to serve as foundational data for developing comprehensive policies that address the global spread of NENTPs.

## 2 Methods

A scoping review and thematic analysis were conducted. The objectives did not include any formal evaluation of research quality; instead, the aim was to broadly synthesize the current literature related to the research objectives, supporting the appropriateness of the scoping review approach (24). As the NENTPs policies reviewed pertain to various countries and populations, evaluating the quality of individual policies was considered to add minimal value. The specific objectives were to (1) explore policy implications associated with new e-cigarette policies and (2) identify commonalities among those policy implications. In this review, NENTPs were novel and emerging nicotine and tobacco products and referred to a wide range of non-combustible or alternative products. These included, but were not limited to, e-cigarettes (including disposable and reusable devices), heated tobacco products (HTPs), nicotine pouches, and other novel smokeless nicotine or tobacco-related products. However, traditional combustible tobacco products, including menthol cigarettes, were explicitly excluded from this definition. While these products differ in their material composition, this review analyzed them collectively based on their common regulatory implications and the implementation actions in the included studies.

### 2.1 Design

A scoping review of the available scientific literature was conducted following the Preferred Reporting Items for Systematic Reviews and Meta-Analyses extension for Scoping Reviews guidelines (25). However, this review was neither registered nor did it have any previously published protocols.

### 2.2 Search strategy

The search strategy was developed based on a literature review aligned with the objectives. It was peer-reviewed by the authors using the “Peer Review of Electronic Search Strategies” framework and executed across the following four databases: PubMed, Embase, Web of Science, and CINAHL to ensure a multidimensional representation of the NENTP regulatory landscape, which intersects clinical medicine, social policy, and behavioral science. The strategy considered the unique syntax, controlled vocabulary, and proximity operators relevant to each database. The primary search strategy utilized descriptors related to NENTPs (e.g., e-cigarette, heated tobacco product) and policy (e.g., regulation, control). The full search strategies for each database are documented in Supplementary Table 1. The most recent search was conducted on March 15, 2025.

### 2.3 Eligibility criteria

Included studies had to report or discuss policies within the context outlined in Table 1, using the Population/Problem, Phenomenon of Interest, Context, and Design framework (26, 27). This format was employed in place of the traditional Population/Problem, Intervention, Comparator, Outcomes, and Design format owing to the qualitative nature of the objectives and characteristics of the phenomenon of interest.

**Table 1:** Research questions in PICo-D format.

Component	Description
Population/problem	Adolescent, adult
Phenomenon of interest	New and emerging nicotine and tobacco product law
Context	Policy implication
Design	Quantitative, qualitative studies

Inclusion and exclusion decisions were made by mutual agreement between the authors. Articles were excluded if they did not address policies within the scope of NENTPs control or focused only on health implications. We included only policies that have been implemented in the real world regarding NENTPs (e.g., electronic nicotine delivery system (ENDS)) and excluded studies of hypothetical bans or policy experiments. No restrictions were imposed on country of origin or publication year; however, searches were limited to English only due to pragmatic reasons. Duplicates were screened using EndNote software.

### 2.4 Reference screening and data extraction

Screening was conducted independently in two phases by two authors, in accordance with the selection criteria. In the first phase, titles and abstracts from the bibliographic search were screened. In the second phase, full texts of studies included in the first phase were reviewed. Data were extracted using Microsoft Excel, with data extraction sheets developed based on a literature review. Variables included authors and publication year, country, policy description, study design, population, main findings, and policy implications. Any discrepancies between the authors were resolved through iterative discussion and a collective re-examination of the eligibility criteria until a complete consensus was reached. Considering the nature of a scoping review, the methodological quality of the included studies was not assessed.

### 2.5 Data synthesis

A thematic framework approach (28) was employed owing to the broad and conceptual nature of the themes addressed in this review. This method was deemed the most suitable for generating an objective synthesis of current perspectives on NENTPs control policies. Themes across policies were synthesized to identify implications for future policy development. Analytical themes were identified through a literature review and coding, from which descriptive themes were developed. Disagreements during the process were resolved through consensus discussions.

## 3 Results

### 3.1 Study selection and characteristics

**Figure 1:**
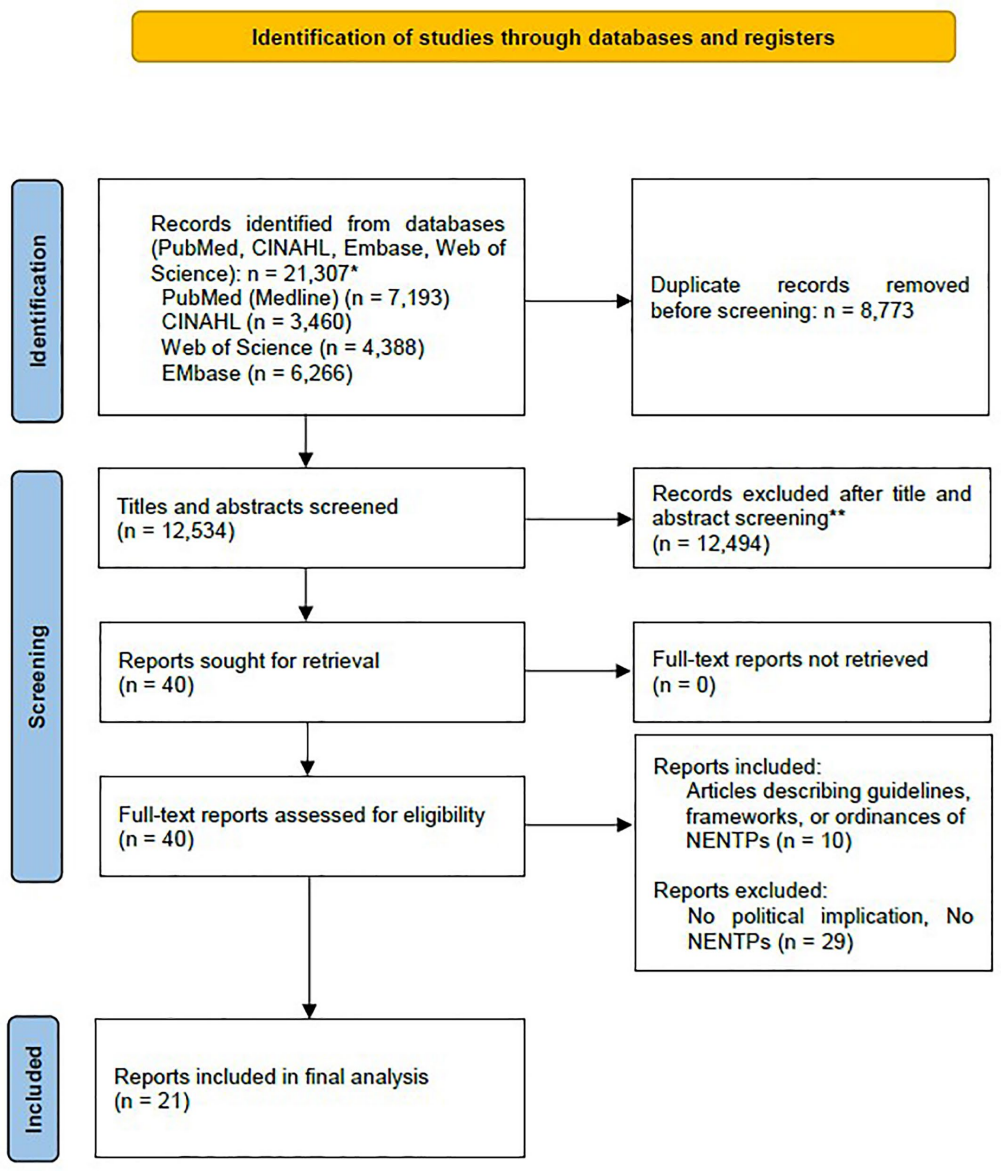
PRISMA diagram of articles included in scoping review, developed with PRISMA flow diagram tool (25).

A total of 21,307 studies were found through the systematic search. Of these, 12,534 remained after removing duplicates. After screening titles and abstracts, 40 studies were selected for full-text review. Out of these, 21 studies met the inclusion criteria and were included in the final analysis. Reasons for exclusion at the full-text stage included articles that focused on health-related implications or were unrelated to NENTPs. Figure 1 illustrated the study selection process.

**Figure 2:**
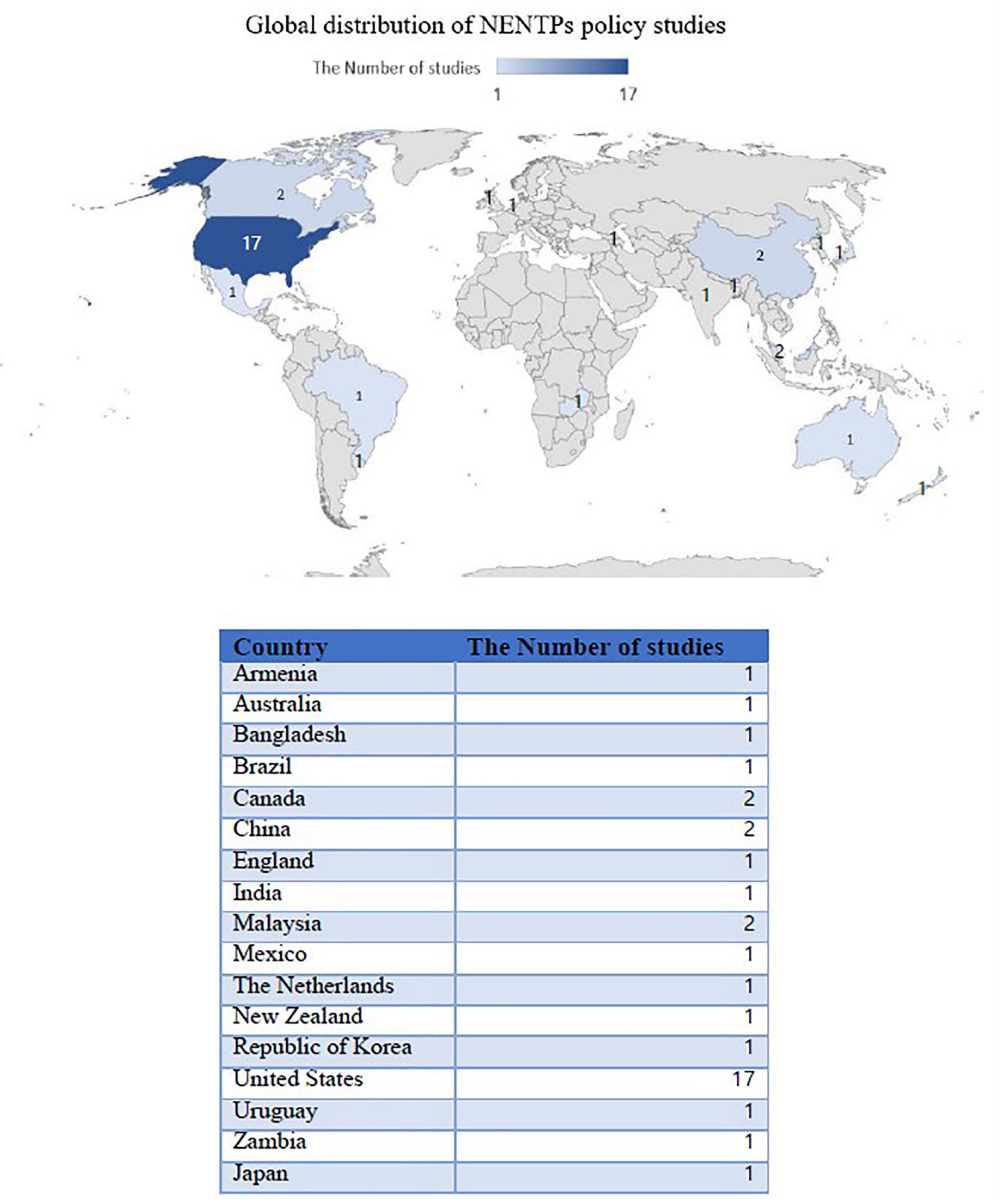
Global distribution of NENTPs’ policy studies.

Among the 21 included studies (Table 2), most employed quantitative designs (*n* = 18), followed by mixed-methods designs (*n* = 1), and systematic review designs (*n* = 2). The majority of studies were conducted in high-income countries, including the United States (*n* = 17), England (*n* = 1), Canada (*n* = 2), China (*n* = 2), and Japan (*n* = 1) (Figure 2). Income classifications were based on data from the World Bank Countries and Economics (29). Additionally, although the studies did not focus on a single product but rather on a variety of products, most studies (19 studies) examined e-cigarettes (ENDS) and nicotine vaping products (NVPs), followed by HTPs (2 studies).

**Table 2:** Characteristics of included studies on policies regulating NENTPs.

No.	Author	Country	Policy description	Study design	Study population
1	Gravely et al. (2019) (4)	Multi-country (14 countries)	Tobacco control policy	Cross-sectional survey	Adult smokers and former smokers
2	Katchmar et al. (2021) (16)	United States	Massachusetts House Bill No.4196	Cross-sectional survey	Retail sales data for tobacco products in Massachusetts and comparison states
3	Wong et al. (2017) (17)	Malaysia	The ban on sales of nicotine e-liquid	Cross-sectional survey	E-cigarette users in Hong Kong
4	Milicic et al. (2018) (30)	Canada	School-based e-cigarette control	Longitudinal survey	Secondary school students in Ontario
5	Han et al. (2023) (31)	United States	Statewide vaping product excise tax policy	Cross-sectional survey	Young adults in the U. S.
6	Schillo et al. (2023) (32)	United States	Flavored tobacco product restrictions	Cross-sectional survey	Adolescents and young adults in Massachusetts and New York
7	Chen et al. (2024) (33)	United States	bans on sales of electronic nicotine delivery systems	Spatial econometric analysis	Population-level data across U. S. states implementing ENDS bans
8	Cheng et al. (2024) (34)	United States	Restrictions on nicotine vaping product sales	Cross-sectional survey	U. S. adults who use nicotine vaping products
9	Togawa et al. (2024) (35)	Japan	Revised smoke-free regulations under the 2020 Health Promotion Act	Longitudinal study	Adults who smoke cigarettes, use HTPs, and do not use any other tobacco products.
10	Agaku et al. (2022) (37)	United States	Federal Tobacco 21 (T21) Law	Cross-sectional survey	Young adults across U. S. states
11	Grigoryan et al. (2023) (38)	Armenia	Indoor and outdoor smoking ban in dining venues	Mixed-methods evaluation (survey and focus group)	Dining venue employees and patrons
12	Fix et al. (2024) (39)	United States	Ban on flavored ENDS products	Longitudinal survey	Adults in New York who use ENDS
13	Yan et al. (2023) (40)	China/ United States	E-cigarette policies	Systematic Review	Not applicable
14	Ali et al. (2022) (41)	United States	Statewide restrictions on flavored e-cigarette sales (2014–2020)	Cross-sectional survey	Retail e-cigarette sales data across U. S. states
15	Brown et al. (2024) (42)	United States	Flavored vaping product sales restriction	Cross-sectional survey	Retailers selling vaping and cigarette products in New York State
16	Zhang et al. (2018) (43)	United States	California’s Tobacco 21 law	Pre-post evaluation using retail data and youth survey	Tobacco retailers and adolescents
17	He et al. (2024) (44)	United States	Excise taxes on cigarettes and vaping products	Longitudinal survey	Current and former smokers
18	Cadham et al. (2022) (23)	United States, India, multiple countries	Restriction on flavored ENDS	Scoping Review	Not applicable
19	Friedman et al. (2024) (36)	United States	ENDS flavor restrictions	Cross-sectional survey	Information Resources Incorporated’s retail sales data
20	Tam et al. (2024) (21)	United States	E-cigarette flavor bans	Cross-sectional survey	US young adults
21	Friedman et al. (2024) (22)	United States	ENDS flavor restrictions	Cross-sectional surveys	US young adults

The thematic analysis identified 33 codes across the 21 studies (Supplementary Table 2), resulting in seven descriptive themes and two analytical themes (Table 3). The two analytical themes were regulatory governance system for the control of NENTPs, exposure reduction at the population level and societal regulation of NENTPs use.

**Table 3:** Descriptive and analytical themes derived from the thematic analysis.

Descriptive theme	Analytical theme
Regulatory spillover control in the national context (4, 16, 23, 30–34) (*n* = 8)	Regulatory Governance System for NENTPs Control
Evidence-informed policy transition and regulation (16, 21, 22, 34–36) (*n* = 6)	
Government monitoring and compliance assurance (17, 31, 37–40) (*n* = 6)	
Product attribute restrictions as an initiation control (21–23, 36, 39) (*n* = 5)	Exposure Reduction at the Population Level and Societal Regulation of NENTPs Use
Regulation of access to retail stores and exposure to youth (4, 37, 41, 43) (*n* = 4)	
Economic restraint through tax increase (16, 31, 44) (*n* = 3)	
Customized communication for policy education (17, 38, 40, 43, 44) (*n* = 5)	

### 3.2 Regulatory governance system for NENTPs control

This analytical theme referred to an integrated governance framework designed to address the technical and market volatility associated with NENTPs. In particular, it described the administrative and legal structures, such as the centralization of regulatory authority, government-led evidence-informed policy systems, and compliance monitoring. This governance system is characterized by three essential descriptive themes—namely, regulatory spillover control in national contexts, evidence-informed policy transition and regulation, and government monitoring and compliance assurance.

#### 3.2.1 Regulatory spillover control in the national context

The first descriptive theme emphasized the need for national-level policies to ensure uniform enforcement and maximize public health outcomes. Several studies highlighted the limitations of fragmented or localized regulations, which can lead to regulatory evasion, spillover effects, and inequitable outcomes (4, 16, 23, 30–34). For example, in response to Massachusetts’ ban on all NVPs, purchases along the state’s border were most pronounced, with sales of tobacco, menthol/mint, and other flavored NVPs in Massachusetts’ border states increasing by more than 50% compared to non-border states (34). Moreover, the full and the flavor ENDS bans had significant positive spatial spillover effects on e-cigarette refill sales in neighboring states without e-cigarette bans (33). Thus, nationwide policy expansion led by governments to reduce e-cigarette use was recommended (16).

#### 3.2.2 Evidence-informed policy transition and regulation

The second descriptive theme highlighted the importance of grounding NENTPs policy development in scientific evidence (16, 21, 22, 34–36) and global guidelines, such as those from the FCTC (35). Under the FCTC, a study (35) called for laws to address indoor and second-hand exposure to new tobacco products. Additionally, another study found that e-cigarette purchases declined while cigarette sales increased after the introduction of an e-cigarette tax in Massachusetts (16), and that more people might consider quitting if flavored e-cigarettes were restricted, although some switch to smoking (21). This highlights the need to evaluate policy outcomes using systematic evidence. To reduce NENTPs’ use among populations, governments should adopt evidence-informed policies (16, 35).

#### 3.2.3 Government monitoring and compliance assurance

Multiple studies emphasized the need for ongoing government oversight to ensure effective enforcement of tobacco control laws (17, 31, 37–40). This includes compliance checks, allocation of enforcement resources, and regulatory oversight. Key areas of concern include black-market activity (17), outdoor smoking violations (38), states or regions without such policy (31), and the regulation of retail and online product distribution (17, 39). Additionally, several studies have identified the government (17, 38) as the primary institutional actor responsible for monitoring policy compliance.

### 3.3 Exposure reduction at the population level and societal regulation of NENTPs use

Four descriptive themes—namely, product attribute restrictions as an initiation control, regulation of access to retail stores and exposure to the youth, economic restraint through tax increase, and customized communication for policy education —were grouped under the second analytical theme, which encompassed regulatory mechanisms such as flavor ban, limited access, economic constraint, and communication.

#### 3.3.1 Product attribute restrictions as an initiation control

Following the implementation of the flavored ENDS ban, use of flavored products declined (21–23, 36, 39). Removing flavors from other tobacco products could be an essential part of a comprehensive approach to lower youth access to and use of flavored e-cigarettes (41). By reducing their attractiveness, this approach could encourage current users to quit and discourage new users, particularly among youth (39). Furthermore, restricting flavored tobacco product sales could reduce flavored product sales, which may lead to fewer initiation and use of tobacco products and associated health problems (32). However, flavored product restrictions could inadvertently push consumers toward combustible tobacco products (22). Thus, complementary strategies are needed to prevent substitution effects and enhance cessation efforts (42).

#### 3.3.2 Regulation of access to retail stores and exposure to youth

The importance of restricting access to NENTPs was underscored, particularly among adolescents and vulnerable populations (4, 37, 41, 43). Higher nicotine vaping product usage in high-income countries with more lenient policies is likely owing to the greater availability and affordability of these products (4). In this regard, policy measures such as mandatory point-of-sale age verification (37) and strengthening legal regulations governing nicotine and vaping products (4) were discussed as important factors in reducing initiation and continued use of NENTPs. The enforcement of the federal Tobacco 21 law was also discussed in terms of its impacts on racial and ethnic subgroups (37).

#### 3.3.3 Economic restraint through tax increase

Several studies introduced the excise tax as a fiscal tool to reduce e-cigarette consumption (16, 31, 44). The frequent and entrenched use of e-cigarettes among young adults may increase their sensitivity to price changes, suggesting that an excise tax on e-cigarette products may be more effective for reducing e-cigarette use (31). Indexing specific cigarette taxes to inflation and unifying taxes across product types were also suggested to maintain the effectiveness of excise taxes, reduce the price competitiveness, and prevent product substitution (44). Meanwhile, the quantity and rate of e-cigarette purchases in Massachusetts remained significantly unchanged after the implementation of the tax, likely due to consumers moving out of the state to avoid the excise tax on e-cigarettes (16), suggesting that nationwide expansion of such policies is needed to reduce e-cigarette use (31).

#### 3.3.4 Customized communication for policy education

The complexity of NENTPs presents a global regulatory challenge, underscoring the importance of tailored communication for policy education that targets both consumers and retailers (17, 38, 40, 43, 44). Policy effectiveness can be enhanced by educating tobacco retailers, youth, and parents, particularly in regions with weak regulatory capacity (40). In a study, nearly 24% of retailers reported “shoulder-tapping” purchases, in which adults purchased cigarettes at the request of youth, highlighting the need for educational initiatives targeting this behavior (43). In Malaysia, where e-cigarettes are sold by pharmacists or physicians, the need for education on the potential benefits of these methods has been reported (17). Ongoing public health education campaigns are also needed to communicate the health risks associated with e-cigarettes (43).

## 4 Discussion

This study analyzed policy suggestions from NENTPs policy-effectiveness research and presented integrated policy implications. A total of 21 studies were reviewed using a scoping review and thematic analysis. Notably, most studies were conducted in high-income countries, indicating a significant lack of research on NENTPs restrictions in low-income countries (4, 23). This finding aligns with a study finding on alternative tobacco and nicotine products (45). In high-income countries, the legal frameworks for implementing the convention tend to be more developed and diverse (12), which may explain the relatively high number of studies evaluating policy effectiveness in these contexts. Given this gap in prior studies, a study demonstrating a positive correlation between NVP use and national income (4) provides further insight that may lead to a lower prioritization of NENTP regulations in low-income countries (12). Therefore, fostering policy research in low-income countries requires greater international attention.

Additionally, most studies were on electronic cigarettes. In contrast, studies on HTPs were relatively limited. The greater accumulation of research on e-cigarettes than on heated tobacco stems from the differences in the timing of their international market introduction. E-cigarettes rapidly expanded in the late 2000s, while heated tobacco products were introduced much later (46). Furthermore, as the surge in youth e-cigarette use has emerged as a major public health concern in several countries, research and surveillance have prioritized e-cigarettes (47).

The first analytical theme concerned the regulatory governance system, encompassing the necessity of national-level policies, evidence-informed policy, and government monitoring. Given that most of the studied articles in this study were conducted in high-income countries, the first analytical theme suggests that NENTPs policy may be difficult to implement in contexts where certain institutional conditions are not met. The second analytical theme concerned regulatory mechanisms for reducing population-level exposure, including restrictions on product attributes, regulation of youth access, economic restraints, and customized education. However, under governance with insufficient government control, even the best exposure-reduction strategies are limited in their effectiveness (17), likely due to blind spots in implementation. In other words, consolidated national-level governance, as an institutional actor, can serve as a prerequisite for the implementation efficacy of sophisticated regulatory mechanisms such as economic restraint. Furthermore, although it was not within the scope of this review, e-cigarettes are sold by pharmacists and registered medical practitioners in Malaysia (17), whereas in the United States, they are sold by retailers (41). Similarly, the classification of NENTPs by country may lead to differences in regulatory intensity and enforcement pathways (48). Therefore, future policy research should consider each country’s unique institutional environment and flexible response strategies tailored to product typologies.

Regarding the necessity of NENTPs policies at the national level, it is critical for mitigating jurisdictional spillover effects across regions (4, 16, 23, 30–34). Just as policy variations within federal states can weaken enforcement and lead to spillover effects, inconsistencies in NENTPs regulations at the international level can create similar vulnerabilities in the global market. Therefore, a unified regulatory governance system is necessary at the multinational level. Meanwhile, governments continue to face significant pressure and legal opposition from the tobacco industry (49), indicating that the nationalization and globalization of such policies remain challenging and underscoring the need to anticipate and counteract tobacco industry strategies. Therefore, advancing NENTPs policies on a global scale also requires proactive international legal preparation based on evidence, rather than relying solely on fragmented, country-specific, or state-level disputes.

This study’s findings showed that NENTPs regulation should be based on evidence. In a study examining the impact of vaping (50), regulatory lag resulted from rapid product modification, and marketing exceeded existing legislation and enforcement capabilities. This regulatory lag may be due to the time lag between the publication of policy research on its effectiveness and regulatory action (51). Considering this regulatory lag, the “evidence” in this study should not be limited to traditional long-term follow-up studies. Instead, adaptive evidence-informed governance (52) is needed that immediately reflects the best available evidence, such as real-time exposure data from social media (53, 54) in policymaking. This approach is crucial for timely regulation without compromising scientific integrity.

Government compliance monitoring serves as a critical policy-driving mechanism. A lack of government oversight of certain products led to regulatory blind spots (17). Moreover, it warns that enforcement practices may unexpectedly affect marginalized states without such a policy (31), underscoring the need for region-specific monitoring strategies. Collectively, these insights suggest that even well-designed policies must be supported by robust enforcement systems and a region-responsive monitoring system to realize their intended public health outcomes. Furthermore, as noted earlier, surveillance of areas such as black markets and online systems (17, 39) is inherently challenging. Therefore, further research is crucial to understand practical barriers and help governments develop effective surveillance strategies.

Restriction of access through youth regulation was also noted. A previous study emphasized the importance of mandatory age verification at the point of sale (37) while another study reported adults over 21 purchasing tobacco products on behalf of minors (43). As such, even in countries with age restrictions or sales bans, adolescents most commonly accessed e-cigarettes through face-to-face transactions, with social sourcing (e.g., from peers) being the most prevalent method (55, 56). These findings suggest that, to implement access-restriction policies effectively, customized strategies are needed that address both adults and youth, particularly with respect to social and peer-based access channels.

Communication inefficiencies in regulatory changes, such as flavor bans, led to confusion among affected communities and, ultimately, regulatory non-compliance (17). Inconsistencies in policy messaging complicate implementation and reduce policy effectiveness (16). To address these challenges, it is essential to develop population-specific communication materials to support ongoing public education campaigns for consumers and retailers (43). These findings suggest that the successful implementation of NENTPs policies requires effective communication strategies.

### 4.1 Implications for policymakers

These findings provide several key implications for policymakers seeking to strengthen NENTPs regulations in an evolving regulatory landscape. First, the effectiveness of NENTPs regulation can be maximized when policies are grounded in clear regulatory governance at the global level, supported by a robust evidence base and compliance monitoring led by governments. Second, the rapidly changing nature of NENTPs and the industry’s adaptive marketing tactics requires agile policy responses at the population level, such as continuous tax increases, product-attribute restrictions, limited retail access, and customized communication. While most included studies focused on e-cigarettes, the policy implications of this study could extend to a broad range of NENTPs that are increasingly facing similar regulatory challenges.

### 4.2 Limitation

Despite the strengths of this thematic analysis based on a scoping review of recent empirical studies, several limitations should be acknowledged. First, although the study adhered to the systematic review procedure, the protocol was not pre-registered. This impacts the evaluation of its methodological transparency. Second, the analysis included only English-language studies due to pragmatic constraints, such as limited resources for high-quality translation, thereby excluding potentially relevant grey literature and non-English policy studies. Additionally, it also relied heavily on high-income countries, which may bias global policy implications and innovations. Therefore, our findings should be interpreted as a foundational framework applicable primarily to similar socio-economic contexts, rather than as a universal model. Third, the policy implications derived from the analysis depended on the availability and clarity of discussion in the original texts and may have been influenced by the authors’ subjective interpretations. Although the two authors of this review independently performed data extraction, caution is advised when interpreting the findings. Fourth, this review aimed to address NENTPs but concentrated primarily on e-cigarettes, excluding other products classified as NETNPs. Future research is recommended to include a specified percentage of articles for each product type within the literature inclusion criteria.

## 5 Conclusion

This study aimed to identify policy recommendations from regulatory evaluation studies of NENTPs through a scoping review and thematic analysis. The results highlight that effective legal regulation of NENTPs requires regulatory governance and regulatory mechanisms, including population-level exposure-reduction strategies. This study is significant because it provides policymakers with timely, consolidated insights by examining shared perspectives among researchers worldwide. Additionally, the findings are expected to support more comprehensive and internationally coordinated efforts to regulate NENTPs.
