# Supplementary material for: A scoping review and thematic analysis of policy implications of novel and emerging nicotine and tobacco products regulation
Source: Front Public Health. 2026 Mar 13;14:1786077. doi: 10.3389/fpubh.2026.1786077 (PMC13066193; doi:10.3389/fpubh.2026.1786077)
Supplement: Supplementary file 1 [file Data_Sheet_1.docx]

Supplementary Table 1. The search strategy

| **No.** | **DB** | **Search Strategy** | **Fields** |
| --- | --- | --- | --- |
| 1 | **PubMed** | ("smoking/legislation and jurisprudence"[MeSH Terms] OR "vaping/legislation and jurisprudence"[MeSH Terms] OR "vaping/prevention and control"[MeSH Terms] OR "tobacco products/legislation and jurisprudence"[MeSH Terms] OR "smoking cessation/legislation and jurisprudence"[MeSH Terms] OR "tobacco smoke pollution/legislation and jurisprudence"[MeSH Terms] OR "tobacco smoke pollution/prevention and control"[MeSH Terms] OR ("tobacco control/legislation and jurisprudence"[MeSH Terms] OR "smoke free policy/legislation and jurisprudence"[MeSH Terms] OR ("Tobacco Control"[Title/Abstract] OR "Smoke-Free Policy"[Title/Abstract] OR "Tobacco Policy"[Title/Abstract] OR "Smoking Policy"[Title/Abstract] OR "Smoking Control Policy"[Title/Abstract] OR "Tobacco Regulation"[Title/Abstract] OR "Smoking Regulation"[Title/Abstract] OR "Tobacco Program"[Title/Abstract] OR "Smoking Ban"[Title/Abstract] OR "Smoking Prohibition"[Title/Abstract]))) AND ("Policy"[MeSH Terms] OR "Government Regulation"[MeSH Terms] OR ("Policy"[Title/Abstract] OR "Government Regulation"[Title/Abstract] OR "Law"[Title/Abstract] OR "Regulation"[Title/Abstract] OR "Control"[Title/Abstract] OR "restrict*"[Title/Abstract] OR "legislat*"[Title/Abstract] OR "Policy Studies"[Title/Abstract] OR "Policy Study"[Title/Abstract] OR "Policy Research"[Title/Abstract])) AND ("Adolescent"[MeSH Terms] OR "Adult"[MeSH Terms] OR "Middle Aged"[MeSH Terms] OR "Adolescent"[Title/Abstract] OR "Adult"[Title/Abstract] OR "Middle Age"[Title/Abstract] OR "Adolescence"[Title/Abstract] OR "youth*"[Title/Abstract] OR "teen*"[Title/Abstract] OR "teenager*"[Title/Abstract] OR "grownup*"[Title/Abstract]) | Title, Abstract |
| 2 | **CINAHL** | (((MH "Smoking+/LJ/PC" OR MH "Tobacco+/LJ" OR MH "Tobacco Products+/LJ" OR MH "Passive Smoking/PC/LJ" OR MH "Electronic Cigarettes/LJ" OR MH "Smoking Cessation/LJ/PC") OR (MH "Tobacco Control/PC/LJ")) OR TI ("Tobacco Control" OR "Smoke-Free Policy" OR "Tobacco Policy" OR "Smoking Policy" OR "Smoking Control Policy" OR "Tobacco Regulation" OR "Smoking Regulation" OR "Tobacco Program" OR "Smoking Ban" OR "Smoking Prohibition") OR AB ("Tobacco Control" OR "Smoke-Free Policy" OR "Tobacco Policy" OR "Smoking Policy" OR "Smoking Control Policy" OR "Tobacco Regulation" OR "Smoking Regulation" OR "Tobacco Program" OR "Smoking Ban" OR "Smoking Prohibition"))) AND ((MH "Policy Studies+/LJ" OR MH "Government Regulations+/LJ" OR TI ("Policy Studies" OR "Government Regulations" OR "Policy Research" OR "Policy Study" OR "Law" OR "Regulation" OR "Control " OR "Restrict*" OR "legislat*") OR AB ("Policy Studies" OR "Government Regulations" OR "Policy Research" OR "Policy Study" OR "Law" OR "Regulation" OR "Control " OR "Restrict*" OR "legislat*")) AND (MH "Adult" OR MH "Adolescence" OR MH "Middle Age" OR Adolescence OR Adult OR "Middle Age" OR Adolescent OR Youth* OR Teen* OR Teenager* OR grownup*) | Title, Abstract |
| 3 | **Embase** | ('tobacco control'/exp OR 'smoking regulation'/exp OR 'smoking ban'/exp OR 'tobacco control':ti,ab,kw OR 'smoking regulation':ti,ab,kw OR 'smoking ban':ti,ab,kw OR 'tobacco policy':ti,ab,kw OR 'smoking policy':ti,ab,kw OR 'smoking law':ti,ab,kw OR 'smoking control policy':ti,ab,kw OR 'tobacco regulation':ti,ab,kw OR 'tobacco program':ti,ab,kw OR 'smoking prohibition':ti,ab,kw) AND ('policy'/exp OR 'government regulation'/exp OR 'law'/exp OR 'control'/exp OR policy:ti,ab,kw OR 'government regulation':ti,ab,kw OR law:ti,ab,kw OR control:ti,ab,kw OR regulation:ti,ab,kw OR restrict*:ti,ab,kw OR legislat*:ti,ab,kw OR 'policy studies':ti,ab,kw OR 'policy study':ti,ab,kw OR 'policy research':ti,ab,kw) AND ('adult'/exp OR 'adolescent'/exp OR adolescent:ti,ab,kw OR adult:ti,ab,kw OR adolescence:ti,ab,kw OR youth*:ti,ab,kw OR teen*:ti,ab,kw OR teenager*:ti,ab,kw OR grownup*:ti,ab,kw) | title, abstract,  Author keywords |
| 4 | **Web of Science** | ((TS=("Tobacco Control" OR "Smoke-Free Policy" OR "Tobacco Policy" OR "Smoking Policy" OR "Smoking Control Policy" OR "Tobacco Regulation" OR "Smoking Regulation" OR "Tobacco Program" OR "Smoking Ban" OR "Smoking Prohibition")) AND TS=(policy OR "government regulation" OR law OR control OR regulation OR restrict* OR legislat* OR 'policy studies' OR "policy study" OR "policy research")) AND TS=(adult OR adolescent OR adolescence OR youth* OR teen* OR teenager* OR grownup*) | TS(Topic):  title, abstract,  author keywords,  and keyword plus. |

Supplementary Table 2. Main findings and policy implications from the selected studies on policies regulating NENTPs

| No. | **Main findings** | **Policy Implications** |
| --- | --- | --- |
| 1 | There was wide variation in awareness and use of nicotine vaping products (NVPs) across countries. Higher levels of awareness were associated with increased prevalence of use and product trial. | “Strict regulation and enforcement of NVP laws in these countries may have limited smokers’ access to these products and/or discouraged smokers from using them.”  “When governments and large public health organizations support using e-cigarettes for smoking cessation, there is an addictive effect on usage rates.” |
| 2 | Massachusetts House Bill No. 4196 was associated with significant reductions in in-state sales, with some evidence of increased cross-border purchases in neighboring states. | “Given this information, it is likely that consumers also traveled outside of Massachusetts to avoid the excise tax on e-cigarettes.”  “If governments wish to reduce e-cigarette use in their populations, they should engage in evidence-based policies that work to lower, not increase, the risk that is incurred by these populations, and design messaging that accurately portrays risk associated with behaviors such as e-cigarette and combustible cigarette use.” |
| 3 | Many users opposed a total ban, citing harm reduction and cessation benefits. Support for regulatory approaches was stronger than for outright prohibition, especially among dual users. | “Authority should look into tightening the regulations of selling nicotine e-liquid amidst zero nicotine e-liquid in shops …obtaining nicotine e-liquid from the black market incurred potential risk such as the uncertainty of the safety and quality of the products.”  “Findings imply the necessity to educate e-cigarette users to understand the underlying benefits of obtaining e-cigarettes from pharmacists and medical practitioners. |
| 4 | The policy significantly reduced students' intentions to initiate e-cigarette use. | “School-level policies banning the use of e-cigarettes on school property may be effective in reducing e-cigarette use (or preventing e-cigarette use) in their current form .” |
| 5 | Excise tax policies on vaping products were linked to reduced usage among young adults. | “Considering that there are still a number of US states that have not implemented the vaping product excise tax policy, wider adoption of such policy across the nation would likely help reduce ENDS use rates.”  “It is likely that frequent and established ENDS use among young adults makes them more sensitive to price changes in ENDS products, which may, in turn, make vaping product excise tax policy more effective in suppressing ENDS use among young adults.”  “Future monitoring for states without such policy is needed since the effects of the policy for these states remain uncovered.” |
| 6 | Flavored vaping product restrictions in New York were associated with decreased use. | “Flavored tobacco sales restrictions decrease sales of flavored products in areas subject to the policy, which may lead to decreased initiation and use of tobacco products and subsequent health effects.” |
| 7 | State-level bans on ENDS (electronic nicotine delivery systems) led to decreased usage within those states but also caused spatial spillover, with increased use reported in neighboring states without such bans. | “ENDS bans would potentially lead to either cross-border purchases or substitutes to unrestricted products.” |
| 8 | Emergency restrictions on NVP sales were associated with increased cross-border purchasing behaviors, suggesting regulatory leakage to states without similar policies. | “Such cross-state border purchases to evade state NVP sales restrictions may alleviate policies’ intended effects on decreased NVP accessibility, decreased NVP use, and improved public health.”  “Policy makers should factor in these purchasing behaviors to evade state sales restrictions when evaluating any future potential policies at the state or local levels.” |
| 9 | The amended HPA in Japan resulted in significant decreases in smoking and HTP use in restaurant and bars. | “Governments should consider regulating indoor HTP use in accordance with the scientific evidence regarding the harmfulness of HTP aerosols. “ |
| 10 | The federal Tobacco 21 (T21) law was linked to a modest reduction in youth tobacco access and use. | “Efforts to increase tobacco retailers' compliance to mandatory age checks are warranted in all communities, but especially among racial and ethnic minority communities for who our findings suggest suboptimal enforcement of state access laws. The equitable and intensified enforcement of the federal T21 law among all racial ethnic subgroups and across all tobacco products may achieve a positive equity impact in reducing all forms of tobacco use among US youths and young adults.” |
| 11 | Employees and patrons reported high awareness and support for indoor and outdoor smoking bans. While indoor smoking declined, compliance with outdoor restrictions was inconsistent. | “The Government of Armenia should enhance the monitoring and enforcement activities.”  “Targeted and tailored awareness-raising activities are needed both for the general public and the hospitality industry.” |
| 12 | Following implementation of the flavored ENDS ban, use of flavored products declined, while use of non-flavored and tobacco-flavored products increased. | “The removal of flavors from other tobacco products (cigarettes, cigars, smokeless tobacco) may result in reduced appeal for these products as well, possibly encouraging some current users to quit and discouraging new users from starting, particularly youth.”  “There appears to be a need for additional understanding of how users are procuring these flavored products. Continued evaluation of how to improve compliance with current restrictions on retail and online sale of these products are critical to understanding the long-term impact on use patterns.” |
| 13 | Flavor regulations reduced youth electronic nicotine product use, and taxes reduced adult use | “Policy impacts would be improved by educating tobacco retailers, youth, parents, as well as creating changes in social environments, especially in areas currently without strong regulatory power.”  “This finding indicates the necessity of enforcement and monitoring.” |
| 14 | Statewide flavor bans were associated with declines in flavored e-cigarette sales, though their effectiveness varied depending on enforcement rigor and policy scope. | “Restrictions on non-tobacco-flavored e-cigarette sales may be an important part of a comprehensive approach to reducing youths' access to and use of flavored e-cigarettes.” |
| 15 | The flavored vaping product sales ban in New York State resulted in significant declines in flavored vape sales. | “Flavored product restrictions may unintentionally shift consumers back to combustible tobacco products. Complementary strategies are needed to prevent substitution effects and reinforce cessation support.” |
| 16 | Following the implementation of the law, youth access to tobacco products declined. Retailer compliance improved, and adolescents reported reduced access to tobacco products through retail outlets. | “E-cigarette users were significantly less likely to agree, suggesting that continued public health education campaigns are needed to communicate the health risks associated with e-cigarettes.” |
| 17 | Taxation played a significant role in influencing switching and quitting behaviors. | “in order to make cigarettes much less affordable than ECs, policymakers will need to continue  to raise cigarette excise taxes, particularly in areas where such taxes have not been increased in recent years. Additionally, to maintain the effectiveness of excise taxes and reduce the affordability of cigarettes, policymakers may need to index specific cigarette taxes with inflation. As the cost of living significantly increased in recent years, it is important for policymakers to adjust excise taxes accordingly to decrease the relative affordability of cigarettes compared to ECs and other consumption goods.”  “This suggests that younger people’s nicotine and tobacco use behaviors may be less established and potentially more responsive to interventions. On the other hand, targeted efforts and innovative policy interventions may be necessary to encourage behavioral changes among older people who smoke or vape.” |
| 18 | Studies on flavored ENDS found 6–39% of stores sold restricted flavored products post-restrictions. Online stores remain a potential source. | “Drawing concrete conclusions on patterns of use from this data is challenging, especially for smaller jurisdictions where individuals can easily travel to areas without restrictions and buy  products online or at exempt retailers.”  “These studies suggest that, with enforcement in place, flavor restrictions substantially reduce the availability of flavored products in mass-market retail stores.”_ |
| 19 | ENDS flavor restrictions lead to substantial drops in total sales, mainly due to declines in flavored ENDS and slight increases in unflavored ENDS. Restrictions also raise sales of combustible cigarettes. | “E-cigarettes could continue to have internalities though, particularly if youth do not anticipate nicotine addiction’s impact on future cessation attempts.”  “Policymakers could also consider pairing a policy that allows more flavors in ENDS with restricting all non-medicinal tobacco product sales to adult-only stores, to reduce internalities among time-inconsistent (or uninformed) youth while allowing adult access to flavors in order to promote smoking cessation.” |
| 20 | About 80% continued vaping despite real-world changes in flavored e-cigarettes. Among exclusive vapers, 12.5% switched to cigarettes. | “This suggests that larger shares of this population might consider quitting if flavored e-cigarettes were made inaccessible, but that larger shares might also consider switching to smoking.” |
| 21 | State ENDS flavor restrictions led to significant drops in daily vaping and rises in daily cigarette smoking. | “Policies that make ENDS more expensive (taxes) or less appealing (flavor restrictions) are likely to increase use of more dangerous combustible cigarettes in this age group (young adults).” |
